# Ligand-Capped Cobalt(II) Multiplies the Value of the Double-Histidine Motif for PCS NMR Studies

**DOI:** 10.1021/jacs.2c12021

**Published:** 2023-02-14

**Authors:** Wenkai Zhu, Darian T. Yang, Angela M. Gronenborn

**Affiliations:** † Department of Structural Biology, University of Pittsburgh, School of Medicine, 3501 Fifth Avenue, Pittsburgh, Pennsylvania 15261, United States; ‡ Department of Chemistry, University of Pittsburgh, Dietrich School of Arts and Sciences, 219 Parkman Avenue, Pittsburgh, Pennsylvania 15260, United States

## Abstract

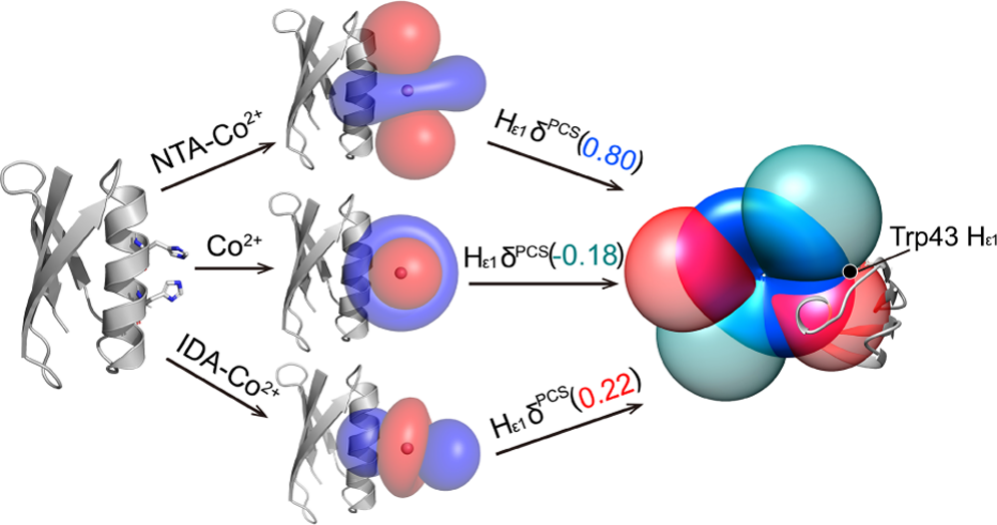

In structural studies by NMR, pseudocontact shifts (PCSs) provide both angular and distance information. For proteins, incorporation of a di-histidine (diHis) motif, coordinated to Co^2+^, has emerged as an important tool to measure PCS. Here, we show that using different Co(II)-chelating ligands, such as nitrilotriacetic acid (NTA) and iminodiacetic acid (IDA), resolves the isosurface ambiguity of Co^2+^-diHis and yields orthogonal PCS data sets with different Δχ-tensors for the same diHis-bearing protein. Importantly, such capping ligands effectively eliminate undesired intermolecular interactions, which can be detrimental to PCS studies. Devising and employing ligand-capping strategies afford versatile and powerful means to obtain multiple orthogonal PCS data sets, significantly extending the use of the diHis motif for structural studies by NMR.

## Introduction

Pseudocontact shifts (PCSs) encode distance and orientation information for the observed nucleus with respect to a paramagnetic center, and PCSs are widely used for protein structure determination, refinement, and conformational studies. In the early days of protein NMR, native metal centers were exploited for chelating paramagnetic ions,^1,2^ while at present, specially designed metal- or lanthanide-binding tags (LBTs) are covalently attached to surface-exposed amino acids, predominantly cysteines, and loaded with the desired paramagnetic ions.^3−5^ In addition, metal-binding peptide motifs, such as those containing two strategically placed histidines (diHis),^6^ or amino-terminal Cu^2+^- and Ni^2+^-binding motifs (ATCUN) first observed in albumin,^7^ have been engineered into proteins to coordinate paramagnetic ions.^8−10^ These latter methodologies are advantageous when native cysteines are essential for structural integrity and protein function. DiHis motifs can be incorporated into α-helices at positions *i* and *i* + 4 and/or β-strands at positions *i* and *i* + 2.^6^ They have been employed successfully for paramagnetic NMR^11^ and electron paramagnetic resonance (EPR) studies,^12−17^ yielding remarkably precise distance information. Recently, the Otting group demonstrated that Co^2+^-diHis generates sizable PCSs owing to its large anisotropic Δχ-tensor within the transition metal series.^8^ However, several practical drawbacks may limit the use of the diHis motif for PCS studies: (i) diHis-coordinated metal ions can cause protein dimerization or aggregation via unfilled coordination sites. Such metal-induced dimerization has been observed for Zn^2+^, Cu^2+^, and Ni^2+^^14,18,19^ and even exploited in the design of metal-templated protein interfaces;^20^ (ii) the choices of different metal ions for PCS studies that coordinate the diHis motif are limited, with only Co^2+^ generating large enough PCSs for structural exploitation;^1^ and (iii) to resolve isosurface ambiguity, it is necessary to place several diHis motifs in different locations of a protein, such that multiple PCS data sets are generated.^21^ This requirement can create a major challenge, since neither the protein structure nor function should be altered in the diHis variants.

**Figure 1:**
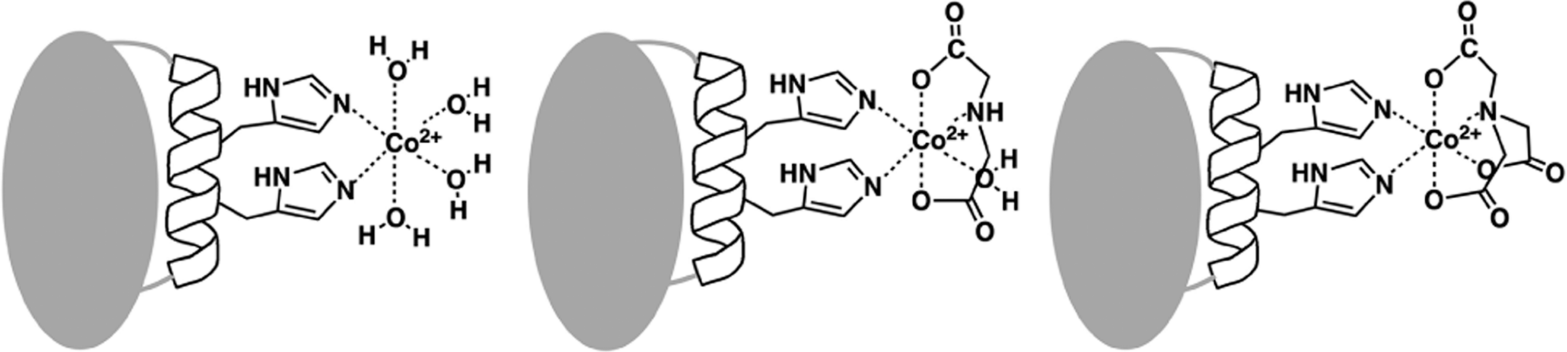
Schematic representation of a diHis motif-bearing protein coordinated to Co^2+^ in the absence and presence of the capping ligands IDA and NTA.

It is well established that the ligand field, i.e., the type and polarizability of the ligand as well as the detailed geometric and electronic nature of the coordinating sphere around the metal ion, plays an important role in modulating the Δχ-tensor properties.^22−25^ In particular, the anisotropy of the magnetic susceptibility tensor, responsible for the amplitude and sign of the PCS, is exquisitely sensitive to structural details in the first coordination sphere around the metal ion.^22−25^ This has been shown clearly for the metal environment in metalloproteins.^24,25^ Herein, we explored the use of different capping ligands to modulate the ligand field of Co^2+^-diHis GB1. We employed tridentate iminodiacetic acid (IDA) and tetradentate nitrilotriacetic acid (NTA) metal chelating groups (Figure 1) and showed that dramatically different Δχ-tensors for the two capped Co^2+^-diHis-bearing GB1 proteins ensued. This allowed us to obtain two extra, independent PCS data sets, in addition to that measured for the uncapped Co^2+^-diHis-bearing protein and unambiguously permitted the positioning of single atoms, such as the NH_ε1_ atom of Trp43 in GB1, at the intersection of three PCS isosurfaces. To further demonstrate the robustness of this methodology, the intersubunit orientation of the HIV-1 capsid C-terminal dimerization domain (CA CTD dimer) was characterized using a PCS data set for the NTA-Co^2+^-diHis (D166H, K170H) CA CTD dimer.

High-spin cobalt(II) has six coordination sites, two of which are occupied by non-hydrogen-bearing nitrogen atoms of the imidazole ring when His residues are placed in *i* and *i* + 4 positions in helices. Therefore, the free coordination sites can be occupied either by water molecules or other ligands. This may cause complications in the exploitation of Co^2+^ for paramagnetic NMR, such as the well-documented coordination of Ni^2+^ and Cu^2+^,^18^ which, dictated by the inner-sphere octahedral or square-planar metal coordination geometry, can lead to *D*_2_-symmetrical tetramers or *C*_3_-symmetrical trimers or *C*_2_-symmetrical dimers.^27^ It therefore seemed prudent to explore the effect of capping ligands for a Co^2+^-diHis protein. This is of particular importance for paramagnetic NMR studies that require a diamagnetic counterpart of the system as a control.

## Results and Discussion

**Figure 2:**
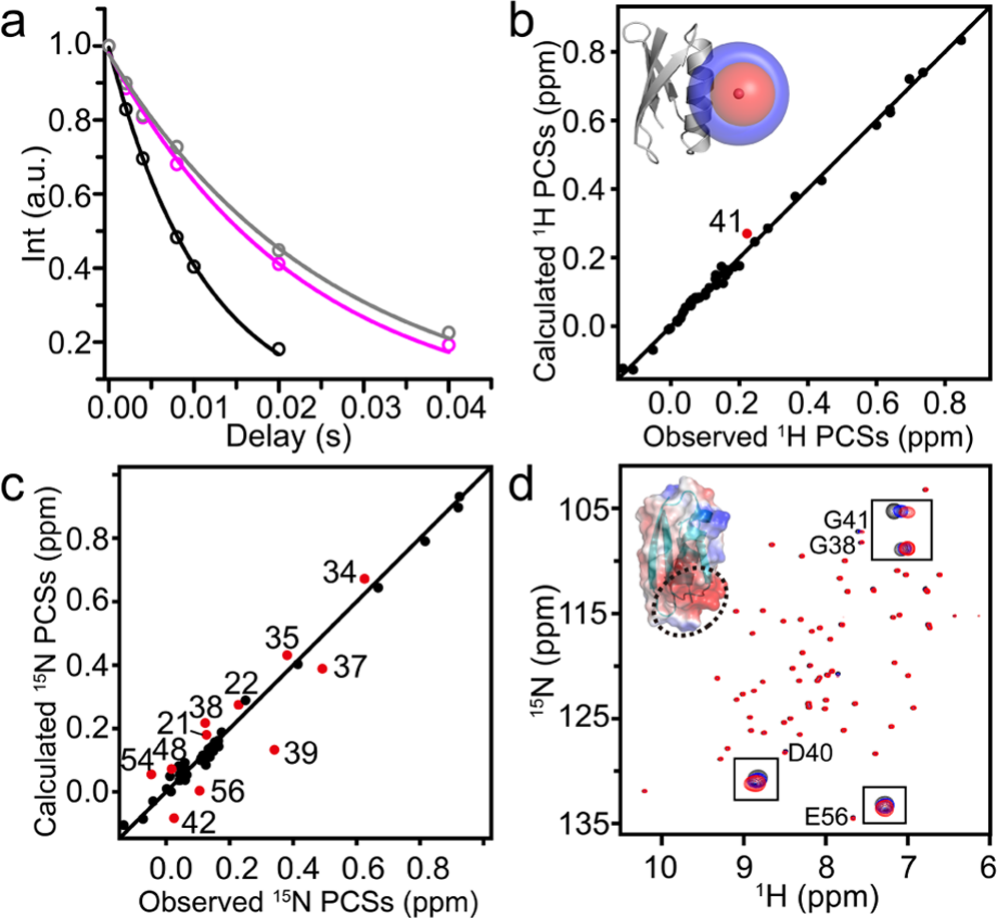
Relaxation and PCS analysis of Me^2+^-diHis GB1. (a) ^19^F *R*_2_ data for diHis GB1 (gray), Zn^2+^-diHis GB1 (black), and Co^2+^-diHis GB1 (magenta). (b, c) Correlation between experimental and calculated (b) ^1^H and (c) ^15^N PCS for Co^2+^-diHis GB1. The PCS isosurfaces for PCSs of ±1.0 ppm, representing the Δχ-tensor, are shown around the Co^2+^ ion on the structure in blue and red, respectively. (d) Superposition of ^1^H–^15^N HSQC spectra of 0.2 mM GB1 without and with CoCl_2_ at 0:1 (black), 2:1 (blue), and 4:1 (red) molar ratios. The region involved in nonspecific interactions with Co^2+^ is indicated by the dotted oval on the GB1 structure model (ribbon embedded into a transparent space-filling surface, color-coded according to the electrostatic potential).

We selected K28H/Q32H GB1, referred to as diHis GB1, which contains a suitable double His motif in its α-helix,^16^ and incorporated a single fluorine at the 5 position in the indole ring of Trp43,^28^ placing the fluorine atom at the center of GB1’s hydrophobic core. ^19^F *R*_2_ relaxation permitted an estimation of the rotational correlation times (τ_c_) for the differently metal-coordinated proteins, since ^19^F *R*_2_ is dominated by the ^19^F chemical shift tensor and is proportional to τ_c_ for isotropic tumbling molecules in the absence of significant internal motion and chemical exchange.^29^ Surprisingly, the ^19^F *R*_2_ value measured for Zn^2+^-diHis GB1 was twice as large as that of non-metal-coordinated diHis GB1, implying that Zn^2+^-diHis GB1 exists as a dimer in solution (Figure 2a and Table S1). This was confirmed by one-dimensional (1D) ^1^H–^15^N TRACT^30^ data (Supporting Information, Figure S1a). This finding is similar to the results of a recent study showing Cu^2+^-induced dimerization of the same diHis GB1 protein by EPR spectroscopy.^19^ For Co^2+^-diHis GB1, on the other hand, ^19^F *R*_2_ is very similar to that for the metal-free diHis GB1 (Figure 2a and Table S1), confirming that Co^2+^ binding does not induce multimerization, in agreement with established coordination properties for Zn^2+^ and Co^2+^.^31^

Given that Zn^2+^-diHis GB1 is dimeric, a perfect diamagnetic control for the Co^2+^-diHis GB1 PCS data is lacking. Irrespective, using the amide ^1^H chemical shifts for Zn^2+^-diHis GB1 as the diamagnetic control yields excellent agreement between measured and back-calculated amide ^1^H PCSs for several different experimentally determined GB1 structures (Figure 2b and Table S2). This indicates that chemical shift changes in ^1^H frequencies of amides, caused by Zn^2+^-induced protein dimerization, are very small compared to the large PCSs generated by Co^2+^. Interestingly, the AlphaFold2^32^ predicted structure of K28H/Q32H GB1 (AF2 diHis GB1) exhibited the best (lowest) quality factor (Table S2), although all of the structures of GB1 are very similar (Supporting Information, Figure S2). We therefore used the AlphaFold2 structural model throughout this study, removing any potential variability caused by differences in methodological details (NMR vs X-ray, different X-ray structures). The PCS-derived Δχ-tensor (Δχ_ax_) is small (Table 1), in agreement with a recent PCS study for ubiquitin and another GB1 variant.^8^ For the amide ^15^N PCS, larger differences between measured and calculated values are observed (Figure 2c and Table 1), and the largest differences between measured and calculated PCSs (red data points in Figure 2b,c) appear to be associated with nonspecific binding of Co^2+^ to diHis GB1 (paramagnetic solvent effect). This was confirmed by carrying out a CoCl_2_ NMR titration with WT GB1 (Figure 2d). Amide resonances that exhibit chemical shift changes upon addition of CoCl_2_ are associated with residues D40, E56, and neighboring amino acids, residing in a negatively charged patch in GB1 (Figure 2d) that appears to weakly bind Co^2+^. Excluding ^15^N PCSs for residues in this negative patch from the analysis results in a significantly improved quality factor (Table S2).

**Table 1:** Δχ-Tensor Parameters for the AF2 diHis GB1 Model Structure Coordinated with Co^2+^, IDA-Co^2+^, and NTA-Co^2+^^a^

	Δχ_ax_	Δχ_rh_		metal positions			Euler angles		
metal ion	10^–32^ m^3^	10^–32^ m^3^	quality factors	*x* (Å)	*y* (Å)	*z* (Å)	α (deg)	β (deg)	γ (deg)
Co^2+^	–3.30 (0.10)	–0.08 (0.03)	0.05	–6.15 (0.14)	1.23 (0.18)	–11.85 (0.10)	103 (1)	61 (1)	21 (44)
IDA-Co^2+^	3.04 (0.06)	0.74 (0.11)	0.04	–6.23 (0.20)	–0.76 (0.32)	–12.27 (0.20)	173 (36)	147 (23)	141 (23)
NTA-Co^2+^	–5.80 (0.34)	–1.33 (0.11)	0.04	–6.43 (0.13)	1.69 (0.12)	–12.02 (0.15)	18 (1)	103 (1)	122 (3)

a The tensor parameters were determined by Paramagpy^26^ with fitting errors shown in the brackets.

To prevent any of the above identified complications associated with intermolecular protein–protein or nonspecific interactions, both Zn^2+^ and Co^2+^ were precomplexed with the tetradentate ligand NTA before addition to diHis GB1. This strategy is commonly used in EPR^12,14^ and PRE^11^ studies with Cu^2+^-diHis as the paramagnetic center. No chemical shift changes in the ^1^H–^15^N HSQC spectrum of WT GB1 were observed upon addition of excess NTA-Co^2+^, confirming that any nonspecific interactions were effectively suppressed by complexation of the paramagnetic ion with NTA (Supporting Information, Figure S3a). Furthermore, ^19^F *R*_2_ data showed that NTA-Zn^2+^/Co^2+^-diHis GB1 exists as a monomer in solution, since the measured ^19^F *R*_2_ value is very close to that of diHis GB1 (Table S1). In addition, using ^19^F relaxation dispersion allowed us to exclude any exchange contribution (*R*_ex_) to *R*_2_ from Zn^2+^ or Co^2+^ binding to diHis GB1 (Supporting Information, Figure S1b). Interestingly, the NTA cap significantly improves binding specificity for Co^2+^ to the diHis motif, albeit at the cost of a reduced affinity. The measured *K*_d_ value for CoCl_2_ binding to diHis GB1 is 21 μM, compared to 128 μM for NTA-Co^2+^ (Supporting Information, Figure S4). This is in agreement with the well-known higher specificity coupled to a lower affinity of NTA-Co^2+^ versus NTA-Zn^2+^ toward histidines.^33^

**Figure 3:**
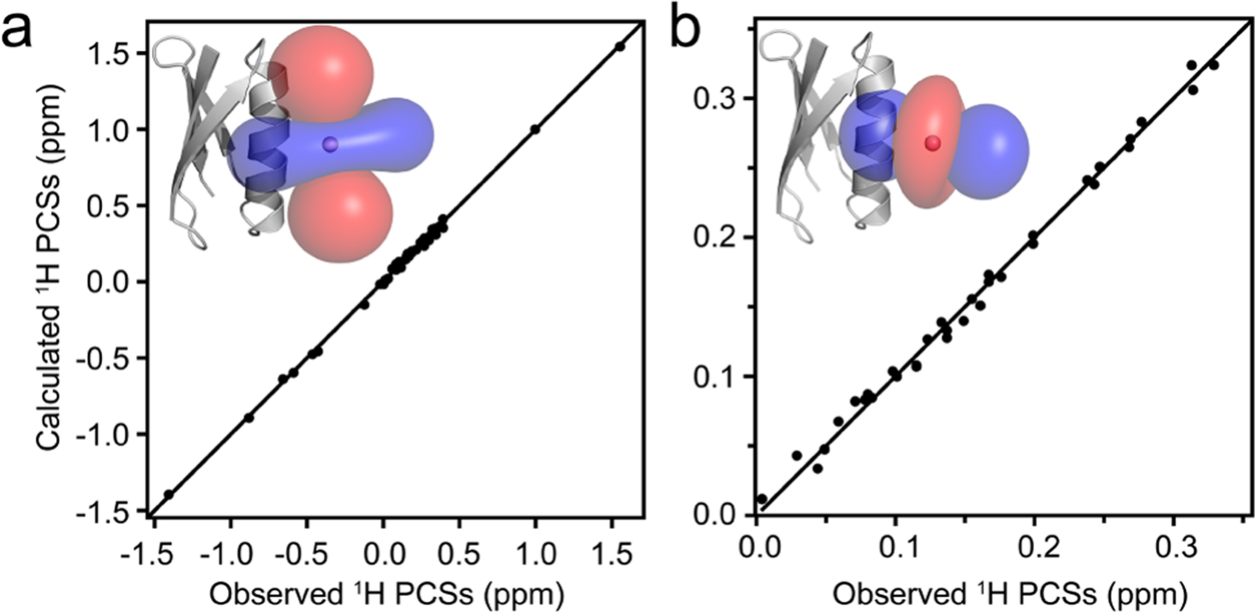
PCS analysis for two capped Co^2+^-diHis GB1 samples. Correlation between experimental and calculated PCSs for (a) NTA-Co^2+^-diHis GB1 and (b) IDA-Co^2+^-diHis GB1. PCS isosurfaces representing the Δχ-tensors for PCSs of ±1.0 ppm are shown around the Co^2+^ ion in the structure models. Note the different scales in (a) and (b).

^1^H and ^15^N PCSs were measured for NTA-Co^2+^-diHis GB1 with NTA-Zn^2+^-diHis GB1 as the control diamagnetic sample. Nearly complete assignments of the ^1^H–^15^N HSQC spectra were obtained for Co^2+^ and NTA-Co^2+^-diHis GB1 using two PCS data sets simultaneously (Supporting Information, Figure S5 and Table S3), exploiting the linear PCS patterns. In contrast to the findings for Co^2+^-diHis GB1, both measured ^1^H and ^15^N PCSs exhibit excellent agreement with predicted ones, based on the AF2 diHis GB1 model (Table 1, Figure 3a, and Supporting Information, Figure S6a), confirming the absence of nonspecific Co^2+^ binding and that correctly matched para-/diamagnetic samples are important. Strikingly, a very different Δχ-tensor was determined for NTA-Co^2+^-diHis GB1 compared to that of Co^2+^-diHis GB1 (see Figures 2b, 3a, and Table 1). This reflects the exquisite sensitivity of the Co^2+^ magnetic susceptibility toward the coordinating ligands. Thus, sufficiently different Δχ-tensors are generated by simply coordinating Co^2+^ with different ligands, resulting in independent PCS data sets. Further confirmation of the importance of the detailed geometry around the metal is provided by the coordination of Co^2+^ with the tridentate IDA ligand, which resulted in a third distinctive Δχ-tensor from the Δχ-tensors for Co^2+^ and NTA-Co^2+^-diHis GB1 as well as better agreement between measured and calculated ^1^H and ^15^N PCSs than seen for Co^2+^-diHis GB1 (Table 1, Figures 3b and S6a). NMR titration for determination of *K*_d_ for IDA-Co^2+^ binding to diHis GB1 provides only an upper limit (212 μM), since excess free IDA can compete with IDA-Co^2+^ binding to diHis GB1 through the formation of an IDA-Co^2+^-IDA complex.^34^ The axial component of Δχ-tensor (Δχ_ax_) for NTA-Co^2+^ is twice that for Co^2+^ alone, while IDA-Co^2+^ possesses a Δχ-tensor of similar size (Δχ_ax_) but of opposite sign of the Co^2+^ alone tensor (Table 1). Importantly, the Δχ-tensors determined for both NTA-Co^2+^ and IDA-Co^2+^ exhibit larger rhombicity (Δχ_rh_) than that for Co^2+^ alone. In addition to the axial (Δχ_ax_) and rhombic (Δχ_rh_) components of the Δχ-tensors, the three Euler angles of the Δχ-tensors are also different with respect to the protein coordinate system (Table 1), reflecting the different molecular environments around the Co^2+^ when coordinated by NTA and IDA. Structurally, capping Co^2+^ by NTA or IDA replaces the coordinated water molecules and dramatically alters the ligand field and environment around Co^2+^. Capped with the tridentate IDA, a single water molecule remains, whereas capped with tetradentate NTA, all four water molecules are replaced, resulting in a less symmetric environment around Co^2+^. This agrees well with the observation that the cobalt ion positions derived from the PCS data are more similar for Co^2+^-diHis GB1 and NTA-Co^2+^-diHis GB1 than those for Co^2+^-diHis GB1 and IDA-Co^2+^-diHis GB1 (Supporting Information, Figure S6b and Table 1). Essentially, three independent PCS data sets were obtained for the three diHis GB1 and Co^2+^ complexes, with all three PCS data sets resulting in excellent correlations between measured and predicted values and small quality factors (Table 1). This suggests that no gross structural changes are induced by complexation of diHis GB1 with Co^2+^ and the two different capping ligands. In addition, both the magnitudes and orientations of RDC-derived Δχ-tensors determined for Co^2+^-diHis GB1 and NTA-Co^2+^-diHis GB1 are comparable to PCS-derived Δχ-tensors (Table S4 and Figure S7), suggesting that Co^2+^ and NTA-Co^2+^ are rigid when coordinated to the diHis motif in GB1.^35^ Similar findings were reported in a previous PCS study using Co^2+^-diHis for ubiquitin.^8^

Previous rotamer modeling studies for NTA-Cu^2+^-diHis used the N_ε1_-H tautomer as a default for both imidazoles that coordinate Cu^2+^.^36^ Unfortunately, it is not clear, a priory, which tautomer is the predominant one for a particular diHis-bearing protein. We therefore collected long-range ^1^H–^15^N HMBC spectra for NTA-Zn^2+^-diHis GB1 (Supporting Information, Figure S8) and established, in combination with density functional theory (DFT)-based optimization, that the N_ε1_-H tautomer for His28 and the N_δ1_-H tautomer for His32 are more favorable (Supporting Information, Figure S9).

**Figure 4:**
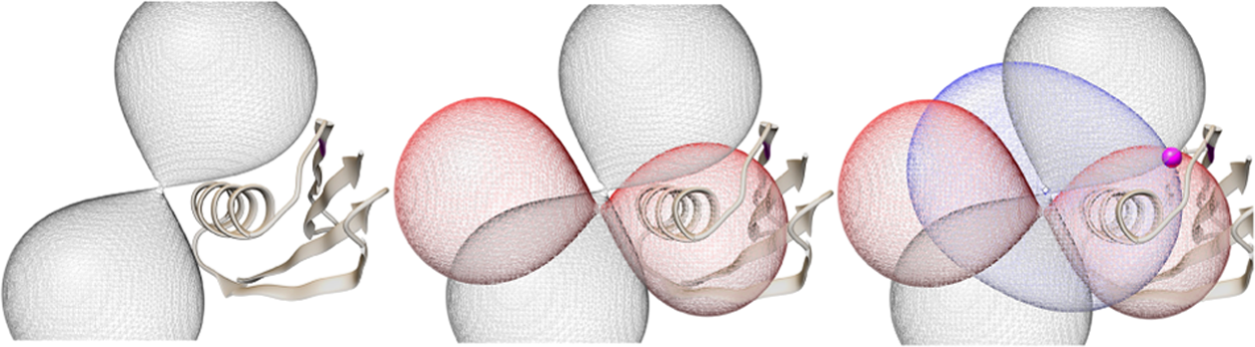
PCS isosurfaces corresponding to the three experimentally determined PCS values for the Trp43 NH_ε1_ resonance of Co^2+^-diHis GB1 (left, −0.18 ppm; gray), plus IDA-Co^2+^-diHis GB1 (middle, +0.22 ppm; red), plus NTA-Co^2+^-diHis GB1 (right, +0.80 ppm; blue). The location of the NH_ε1_ atom of the Trp43 indole (magenta ball) is uniquely defined by the intersection point of the three PCS isosurfaces and the overall protein model.

To demonstrate the orthogonality and advantage of the three PCS data sets for Co^2+^-diHis GB1, IDA-Co^2+^-diHis GB1, and NTA-Co^2+^-diHis GB1, ^1^H PCSs for the Trp43 NH_ε1_ atom were measured. From these data, it is possible to unambiguously locate the position of the NH_ε1_ atom at one of the intersections of the three PCS isosurfaces, and the plausible localization volume is 0.7 Å^3^ assuming a PCS RMSD of 0.02 ppm (Figures 4 and S10). Only one of the four intersecting positions is compatible with the covalent geometry imposed by the structure. This finding agrees well with the results of a recent simulation study, suggesting that the number of tags is more important than the number of tagging sites for structure determination by PCSs.^37^

**Figure 5:**
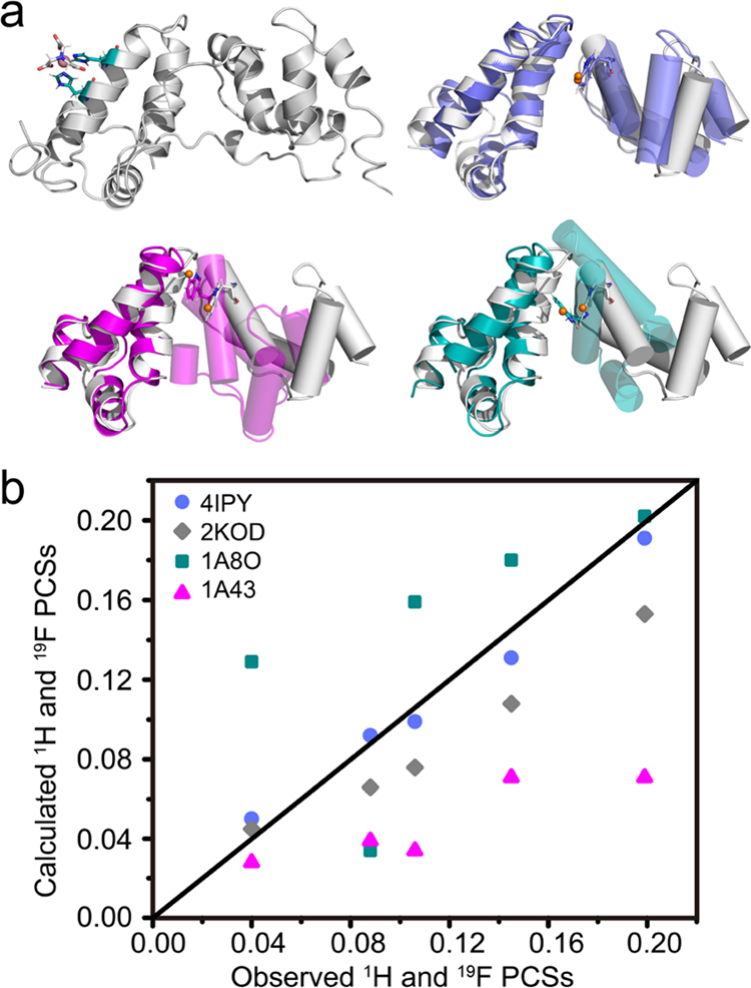
CA CTD dimer structures illustrating different intersubunit orientations in crystals and in solution. (a) Structure model of the NTA-Co^2+^-diHis CA CTD dimer based on the 2KOD NMR structure (gray) and best-fit superpositions of X-ray structures 1A8O (blue), 1A43 (magenta), 4IPY (teal), and the 2KOD NMR structure (gray). One subunit of the CA CTD dimer was the best fit (shown in ribbon representation), and the helices in the other subunit are shown as cylinders. 7F-Trp184 is shown in stick representation with the F atom depicted as an orange sphere. (b) Correlation between the experimental and calculated intersubunit ^1^H and ^19^F PCSs of Trp184 for the different structures in (a) using the same color code.

To apply the above findings to a more complex system, we used the HIV-1 capsid CTD (CA CTD) dimer^38^ for PCS measurements using Co^2+^ as the paramagnetic ion. Previously, numerous distinct quaternary arrangements were observed in different structures of the CA CTD dimer, and interdomain orientations are noticeably different between NMR^39^ and crystal structures, as well as between different crystal structures^38,40−42^ (Figure 5a). A diHis motif was engineered in one of the solvent-exposed α-helices in the CA CTD dimer (D166H, K170H) and a fluorine atom was introduced into the indole ring of Trp184 at position 7. This position was selected, since the intra- and intermonomer distances between His170 Nε and the 7 position of the Trp184 indole ring are distinctly different for the diverse dimer structures. Unfortunately, addition of Co^2+^ to the diHis CA CTD dimer resulted in large PRE effects and only small chemical shifts (Supporting Information, Figure S11), similar to results obtained with the unnatural cobalt(II)-chelating amino acid BpyAla.^43^ Gratifyingly, Co^2+^-induced CA CTD dimer oligomerization was abolished using the NTA capping ligand, and large PCSs were measured for the NTA-Co^2+^-diHis CA CTD dimer (Supporting Information, Figure S12).

To differentiate between intra- and intersubunit PCSs for the CA CTD dimer, a mixed isotopically labeled dimer strategy was employed, comprising ^15^N or ^14^N proteins (Supporting Information, Figure S13). Δχ-Tensor parameters for Co^2+^ in different CA CTD dimer structures were derived using intrasubunit PCSs (Supporting Information, Figure S14, Tables S5 and S6), and these parameters were used to back-calculate the intersubunit PCSs for Trp184 (Table S7). For intersubunit PCSs, only the NMR structure (PDB id: 2KOD) and one of the crystal structures (PDB id: 4IPY) provided predicted PCSs that are in good agreement with the experimentally measured ones (Figure 5b). As noted previously,^44^ two different conformations, D1 and D2, are observed for the CA CTD dimer in solution, in both 1D ^1^H and ^19^F spectra, at 85 and 15% relative populations, respectively (Supporting Information, Figure S12b). Interestingly, D2 exhibits a larger ^1^H (NH_ε1_) PCS than D1 (Supporting Information, Figure S11b), suggesting that different intersubunit orientations are present, with the NH_ε1_ atom in D2 located closer to the cobalt ion than that in the D1 conformation.

## Conclusions

In summary, our results illustrate that differently capped cobalt ions in diHis-bearing proteins provide independent metal Δχ-tensors and generate orthogonal PCS data sets. In addition, the presence of capping ligands effectively prevents metal coordination-induced protein association between diHis motif-bearing proteins, which is detrimental to PCS studies. Together, our ligand-capping strategy significantly broadens the applicability of diHis motifs for PCS measurements in proteins and opens new opportunities for PCS-based structure determination.

## Supporting Information Available

Sample preparation; experimental procedures; and NMR data analysis (PDF)
