# Supplementary material for: Ligand-Capped Cobalt(II) Multiplies the Value of the Double-Histidine Motif for PCS NMR Studies
Source: J Am Chem Soc. 2023 Feb 14;145(8):4564–9. doi: 10.1021/jacs.2c12021 (PMC10032564; doi:10.1021/jacs.2c12021)
Supplement: Supplementary file 1 — ja2c12021_si_001.pdf [file ja2c12021_si_001.pdf]

Ligand-capped Cobalt(II) Multiplies the Value of the Double-Histidine Motif for PCS NMR Studies

Wenkai Zhu[a], Darian T. Yang[a,b], Angela M. Gronenborn[a,b]*

[a] Department of Structural Biology, University of Pittsburgh School of Medicine, 3501 Fifth Ave., Pittsburgh, PA 15261, United States

[b] Department of Chemistry, University of Pittsburgh Dietrich School of Arts and Sciences, 219 Parkman Ave., Pittsburgh, PA 15260, United States

*amg100@pitt.edu

Table of Contents

1. **Experimental Procedures 2-5**

Protein expression and purification 3

Preparation of protein-metal complexes 3

NMR spectroscopy 3

^{19}F R_2 and relaxation dispersion measurements and analysis 4

PCS tensor fitting and error analysis 4

Computational modelling of protein-metal complex 4

2. **Supplementary Figures and Tables 6-23**

Figure S1. ^{1}H-^{15}N TRACT and ^{19}F R_2 relaxation dispersion data for Zn^{2+} coordinated diHis GB1 6

Figure S2. Superpositions of different GB1 structural models 7

Figure S3. NTA-Co^{2+} NMR titration experiment for GB1 and ^{19}F R_2 measured fro NTA-Zn^{2+}-diHis GB1. 8

Figure S4. NTA-Co^{2+} and Co^{2+} NMR titration experiment for diHis GB1 9

Figure S5. ^{1}H-^{15}N HSQC spectra for NTA-Zn^{2+}, Co^{2+}, IDA-Co^{2+} or NTA-Co^{2+} coordinated diHis GB1 10

Figure S6. ^{15}N PCS analysis for NTA-Co^{2+}-diHis GB1 and cobalt(II) positions for free and capped metal ions 11

Figure S7. Correlation between calculated and measured $^{1}D_{HN}$ RDCs for Co^{2+} and NTA-Co^{2+} coordinated diHis GB1 12

Figure S8. Long-range ^{1}H-^{15}N HMBC spectra of NTA-Zn^{2+}-diHis GB1 at different pHs 13

Figure S9. DFT-optimized AF2 diHis GB1 structure coordinated with NTA-Zn^{2+} 14

Figure S10. Localization space of $NH_{\varepsilon 1}$ atom of Trp43 in AF2 diHis GB1 15

Figure S11. Titration of diHis HIV CA CTD dimer with $CoCl_2$ 16

Figure S12. Titration of diHis HIV CA CTD dimer with NTA-Co^{2+} 17

Figure S13. Schematic depiction of mixed labeled diHis CA CTD dimers for intra- and inter-subunit PCS measurements 18

Figure S14. PCS iso-surfaces of NTA-Co^{2+} coordinated diHis CA CTD 19

Table S1. ^{19}F R_2 rates 20

Table S2. $\Delta\chi$-tensor parameters for Co^{2+}-diHis GB1 21

Table S3. Amide ^{1}H and ^{15}N PCSs for Co^{2+}, NTA-Co^{2+} and IDA-Co^{2+} coordinated diHis GB1 22

Table S4. Comparison of $^{1}D_{HN}$ RDCs and ^{1}H PCSs derived $\Delta\chi$-tensors for Co^{2+} and NTA-Co^{2+} coordinated diHis GB1 23

Table S5. Intra-/inter-subunit amide ^{1}H PCSs for mixed labeled CA CTD dimers 24

Table S6. $\Delta\chi$-tensor parameters from intra-subunit ^{1}H PCSs for mixed labeled CA CTD dimers 25

Table S7. Experimental and calculated ^{1}H and ^{19}F PCSs for Trp184 in CA CTD dimer 26

3. **References 27-28**

Protein expression and purification

Coding sequences for diHis GB1 (T2Q, K28H, Q32H) and diHis CA CTD dimer (144-231, D166H/K170H) were inserted into pET11a and pET41 vectors, respectively. DiHis GB1 and diHis CA CTD dimer were expressed in BL21 star (DE3) cells in M9 medium containing 1g/L $^{15}NH_4Cl$. For 5F-Trp or 7F-Trp labeling, 20 mg/L of 5F or 7F-indole (Sigma-Aldrich, St. Louis, MO, USA) were added at OD_{600} = 0.6, respectively, followed by induction after 20 mins with 0.5 mM isopropyl β-D-thiogalactopyranoside (IPTG) (Sigma-Aldrich, St. Louis, MO, USA) and subsequent growth at 18 °C for 16 h. Cells were harvested by centrifugation at 4000 × g for 10 mins and lysed by sonication for 10 mins (2s on and 3s off with 50% power level) in 50 mL QA buffer (20 mM Tris, pH 8.0). The cell lysate was clarified by centrifugation at 18,000g for 30 mins and the supernatant was fractionated on a HP Q column (GE Healthcare, Chicago, IL), followed by gel filtration on a Superdex 75 column (GE Healthcare, Chicago, IL) equilibrated in NMR buffer (20 mM HEPES, 150 mM NaCl, 0.2% NaN_3, pH 7.0). DiHis CA CTD dimer (144-231) was expressed and purified as described previously[1]. In brief, the cell lysate was subjected to fractionation over a HP SP column, followed by gel filtration over a Superdex 75 column. The purity and molecular masses of the proteins were confirmed by SDS-PAGE and ESI mass spectroscopy, respectively.

Preparation of protein-metal complexes

NTA (nitrilotriacetic acid) and IDA (iminodiacetic acid) were premixed with 100 mM $ZnSO_4$, $CoCl_2$ in NMR buffer at ratios of 1.5:1 and 10:1. The pH of the solutions with the metal complexes was adjusted to 7.0 using 10 M NaOH or 37 % HCl to minimize pH changes during the titrations. To measure intra-/inter-subunit PCSs for the CA CTD dimer, mixed isotopically labeled proteins were used. Two samples were prepared: 10 equivalents of non-labeled, natural abundance diHis CA CTD dimer and one equivalent of ^{15}N, 7F-Trp WT CA CTD dimer were mixed as well as vice versa. This allowed measurement of only intra- or inter-subunit PCSs.

NMR spectroscopy

All ^{19}F spectra were recorded on a 14.1 T Bruker AVANCE spectrometer, equipped with a CP TXO F/C-H-D triple-resonance, z-axis gradient cryoprobe at 283 K. ^{19}F chemical shifts were referenced with respect to trifluoracetic acid. ^{19}F spectra were collected with 4,096 data points and a spectral width of 20 ppm using a recycle delay of 1.5 s. The carrier frequency was set to -123 ppm and -133 ppm for 5F-Trp diHis GB1 and 7F-Trp diHis CA-CTD dimer, respectively. ^{1}H-^{15}N HSQC spectra were recorded for diHis GB1 coordinated with Co^{2+}/Zn^{2+}, NTA-Co^{2+}/Zn^{2+} and IDA-Co^{2+}/Zn^{2+} and diHis CA CTD dimer as well as two mixed CTD dimers (see above) coordinated with Co^{2+}/Zn^{2+}, NTA-Co^{2+}/Zn^{2+} with an interscan delay of 1s and 128 complex points in ^{15}N dimension for the ^{1}H PCS measurements. For long-range ^{1}H-^{15}N HMBC spectra, the ^{15}N carrier frequency was centered at 220 ppm and the J coupling constant was optimized to 22.7 Hz for the detection of histidine imidazole cross-peaks. Spectra were recorded with 200 scans and 90 complex points in the ^{15}N dimension. ^{19}F NMR titrations were performed to determine the binding affinities (K_d) between Co^{2+} or NTA-Co^{2+} and diHis GB1. K_d values were extracted from curve fitting of the bound and free populations as a function of the ratio (r) of protein and ligand concentration:

$$f_B=\frac{1}{2}\times(\left(r+1+\frac{K_d}{c}\right)-\sqrt{\left(\left(r+1+\frac{K_d}{c}\right)^2-4r\right)});f_A=1-f_B$$

f_B and f_A denote the bound and free fraction of the protein during the titration and were calculated from the respective integrated peak areas in the ^{19}F spectra. c is the total protein concentration (200 μM) used in NMR titration experiments and was fixed for curve fitting. The K_d between IDA-Co^{2+} and diHis GB1 was calculated to be 212 μM, based on the relative population of diHis GB1 and IDA-Co^{2+}-diHis GB1after adding 0.5-eq IDA-Co^{2+} (mixed at 5:1), as more IDA ligand will be present at higher molar ratio of IDA (5:1) to diHis GB1 and compete off IDA-Co^{2+} binding with diHis GB1.

Backbone amide $^{1}D_{HN}$ RDCs were measured at 600 MHz by IPAP-HSQC experiments[2] with 1024 × 512 data points. Data were processed in Topspin and analyzed in NMRFAM-Sparky[3], and the alignment tensors A were determined using AF2 diHis GB1 structure in Paramagpy[4] and converted to $\Delta\chi_{ax,rh}$ values according to the equation below:

$$\Delta\chi_{ax,rh}=\frac{15\mu_0 k_B T}{B_0^2}A_{ax,rh}$$

where B_0 is the strength of the magnetic field, μ_0 the vacuum permeability, k_B the Boltzmann constant and T the temperature.

^{19}F R_2 and relaxation dispersion measurements and analysis

^{19}F R_1 and R_2 rates were measured by inversion recovery[5] and CPMG[6-7], respectively, using a recycle delay of 2 s. Data processing and analysis were performed in Topspin (Bruker) and MestReNova. R_2 Relaxation rates were obtained by fitting the intensity changes to single exponential functions ($I(t) = I_0 * \exp(-R_2 * t)$). ^{19}F R_2 relaxation dispersion (^{19}F RD) experiments were performed using a modified constant-time 1D CPMG pulse sequence with different π pulse frequencies. As a control, a ^{19}F RD experiment was collected for 5F-indole dissolved in glycerol which exhibited flat dispersion curve (data not shown). Repeated data points for same relaxation delay were collected to estimate R_2 errors. The ^{19}F peak for diHis GB1 shifts continuously during titration with Zn^{2+} and NTA- Zn^{2+} suggesting that the binding of Zn^{2+} and NTA-Zn^{2+} is in fast exchange on the chemical shift scale. Fits of the data to a two-state fast exchange model[8] allowed extraction of $R_{2,0}$ and R_{ex}. $R_{2,0}$ values were used to estimate τ_c, also extracted from 1D ^{1}H-^{15}N TRACT experiments[9].

PCSs tensor fitting and error analysis

^{1}H and ^{15}N PCSs were determined by taking the ^{1}H and ^{15}N chemical shift differences recorded for paramagnetic and diamagnetic protein samples, respectively. Backbone assignments for diHis GB1 were transferred from GB1 and unambiguous assignments were obtained by 3D ^{15}N NOESY-HSQC experiment. Paramagpy[4] was used for fitting $\Delta\chi$-tensor parameters and the uncertainties of tensor parameters were determined by randomly omitting 10% input PCSs with 200 iterations. Protons were added for structural models in this study using Bax's webserver (https://spin.niddk.nih.gov/bax/nmrserver/pdbutil/sa.html). The localization space of $NH_{\varepsilon 1}$ atom of Trp43 in AF2 diHis GB1 was calculated using the script (pcs_fit_atom.py) implemented in Paramagpy as published previously[4,10]. The errors of $\Delta\chi$-tensor were determined from 20 iterations of $\Delta\chi$-tensor fit in which 20% ^{1}H PCSs were discarded randomly. The map size, map density and RMSD contour level in the script was set to be 20.0 Å, 4 points/Å and 0.02 ppm.

Computational modelling of protein-metal complex

Quantum mechanical geometry optimizations were carried out for the NTA-Zn^{2+} diHis coordination complex. The histidine coordinates were taken from residues 28 and 32 of the AF2 diHis GB1 model and truncated to contain only the histidine sidechains, including C_α and C_β atoms. The initial NTA-Zn^{2+} coordinates were taken from the crystal structure[11]. The diHis motif with C_α and C_β atoms of the truncated histidine residues, constrained to fixed positions, was combined with NTA-Zn^{2+} and energy minimized in Avogadro[12] to form a starting structure using the following histidine coordination states: the $N_{\delta 1}$-H tautomer for His32 and $N_{\varepsilon 1}$-H tautomer for His28, the $N_{\varepsilon 1}$-H tautomer for His32 and $N_{\delta 1}$-H tautomer for His28, and the $N_{\delta 1}$-H tautomer for both His32 and His 28.

DFT-based geometry optimization was performed in ORCA 5.0[13] using the BP86 functional[14], the def2-SVP double-zeta basis set[15], the RI-J approximation[16], and DFT-D3 dispersion correction[17]. The Zn^{2+} metal ion was treated with the def2-TZVP triple-zeta basis set[18]. To account for solvation, the SMD solvent model was implemented, which includes both polar and nonpolar solvation components[19]. To maintain the backbone geometry of diHis-GB1, C_α and C_β atoms of the truncated histidine residues were fixed to their initial positions. The resulting geometry optimized structures indicated that for NTA-Zn^{2+}, feasible geometries without breaking the coordination bond from one of the histidine residues were attained for the following diHis motif conformations: the $N_{\delta 1}$-H tautomer for both His32 and His28, and the $N_{\delta 1}$-H tautomer for His32 and $N_{\varepsilon 1}$-H tautomer for His28. From long-range ^{1}H-^{15}N HMBC spectra of the NTA-Zn^{2+}-diHis GB1 sample (Supplementary Figure 7), the coordination complex with a $N_{\delta 1}$-H tautomer for both His32 and His28 could confidently be eliminated, thus leaving the structural candidate with $N_{\delta 1}$-H His32 and $N_{\varepsilon 1}$-H His28 for the diHis NTA-Zn^{2+} complex as the sole option (Supplementary Figure 8).

Models of the diHis CA CTD dimer were initially generated from the NMR structure[1] (PDB ID: 2KOD). For the diHis CA CTD dimer, the D166H and K170H substitutions and the fluorine atom at position 7 of Trp184 were built in Chimera[20]. The initial NTA-Co^{2+} coordinates were taken from the crystal structure[21] and the torsion angles of the histidine residues of the diHis motif were adjusted to match those of the DFT optimized diHis NTA-Cu^{2+} structures[22]. Simulations of CA protein

systems were carried out using the PMEMD module in the AMBER 20 software package[23]. Standard protein residues as well as non-standard fluorinated residues[24] were treated using the ff15ipq force field[25]. The GAFF 2 forcefield[26] was used for NTA ligand parameters and AM1-BCC partial atomic charges for the ligands were calculated using Antechamber[27]. The Co^{2+} ion in each system was treated using a 12-6-4 LJ-type non-bonded model[28]. Each system was solvated in a truncated octahedral box of explicit SPC/E_b[29] water molecules with at least a 12 Å clearance between the protein and the edges of the box. All systems with unpaired charges were first neutralized with Na^+ or Cl^- ions, treated with Joung and Cheatham ion parameters[30], before saturating with enough Na^+ and Cl^- ions to reach a 150 mM NaCl concentration. Protonation states for ionizable residues were adjusted to represent the major species present at pH 7.0. Each system was subjected to energy minimization followed by a two-stage solvent equilibration. In the first equilibration stage, a 20 ps simulation was carried out at constant volume and temperature in the presence of solute heavy-atom positional restraints using a harmonic potential with a force constant of 1 kcal/(mol Å^2). In the second stage, a 1 ns simulation was carried out at constant temperature and pressure using the same harmonic positional restraints. Temperatures were maintained at 283K using a Langevin thermostat with a frictional constant of 1 ps^{-1}, while pressure was maintained at 1 atm using a Monte Carlo barostat with 100 fs between system volume changes. Van der Waals and short-range electrostatic interactions were truncated at 10 Å, while long-range electrostatic interactions were calculated using the particle mesh Ewald method[31]. To enable a 2 fs time step, all CH and NH bonds were constrained to their equilibrium values using the SHAKE algorithm[32]. The structure models of AlphaFold2 diHis GB1 and NTA-Zn^{2+} coordinated diHis GB1 as well as the NTA-Co^{2+} coordinated diHis CA CTD model are available from the authors upon request or can be downloaded from the Github website (https://github.com/darianyang/gb1-pcs).

Supplementary Figures, Scheme and Tables

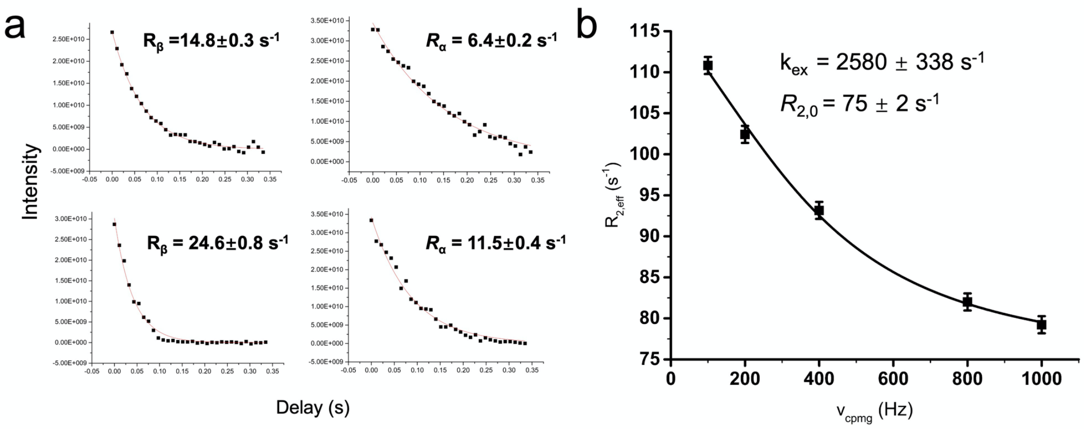

Figure S1. (a) 1D ^{1}H-^{15}N TRACT data for 0.2 mM diHis GB1 (top panel) and in the presence of 4 mM $ZnSO_4$ (bottom panel). (b) ^{19}F R_2 data from a relaxation dispersion experiment recorded for 0.2 mM diHis GB1 in the presence of 4 mM $ZnSO_4$.

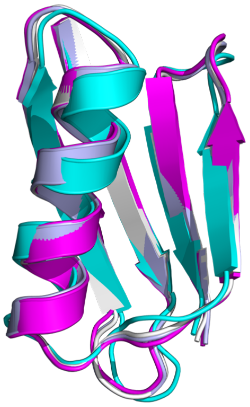

Figure S2. Superpositions of different GB1 structural models: AF2 diHis GB1 (white), NMR structure (PDB id: 2GB1, cyan) and crystal structures (PDB id: 2QMT, light blue; PDB id: 1PGA, magenta). The atomic rmsd values for backbone atoms between AF2 diHis GB1 and 2GB1, 2QMT, 1PGA are 0.93, 0.40 and 0.30 Å, respectively.

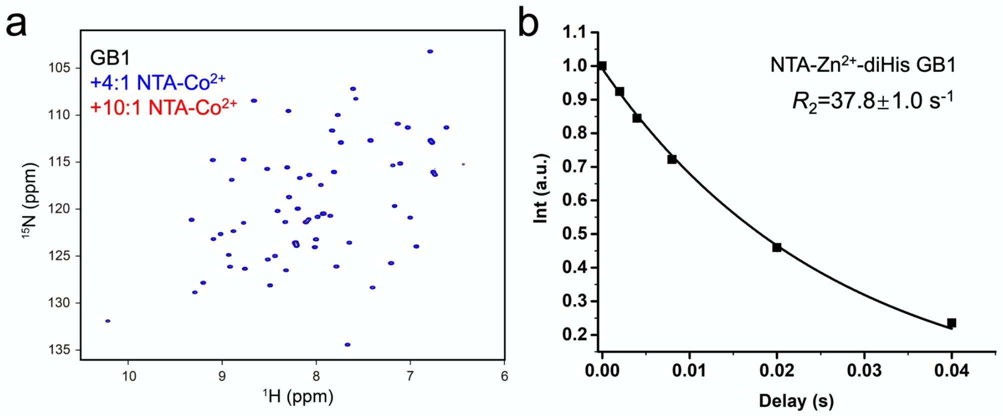

Figure S3. (a) Superposition of the ^{1}H,^{15}N HSQC spectra of GB1 (0.2 mM in 20 mM HEPES buffer, 150 mM NaCl, pH 7.0) at 283K without (black) and with NTA-Co^{2+} at ratios of 4:1 (blue) and 10:1 (red), respectively. (b) Resonance intensities of the ^{19}F signal and R_2 value for diHis GB1 (0.2mM) in the presence of 2mM NTA-Zn^{2+} at 283K in the same buffer as in (a).

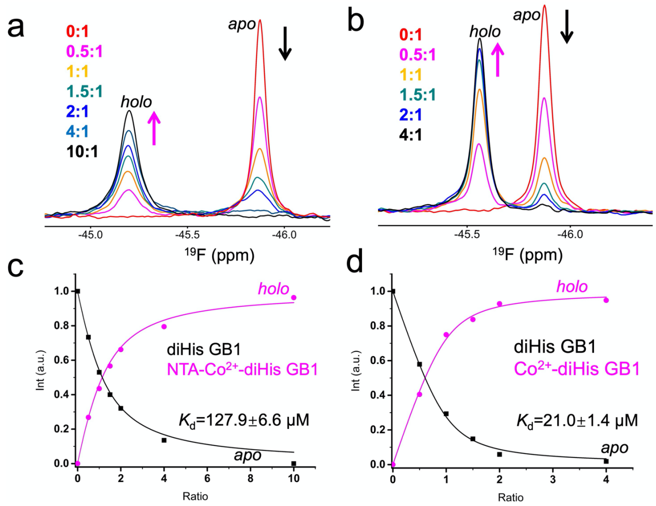

Figure S4. Titration of diHis GB1 (200 μM) with increasing amounts of NTA-Co^{2+} (mixed at 1.5:1 ratio) and Co^{2+}. Superposition of 1D ^{19}F spectra for diHis GB1 in the absence (red) and presence of NTA-Co^{2+} (a) and Co^{2+} (b) for indicated molar ratios. The peaks corresponding to *apo* and *holo* diHis GB1 were integrated separately and fitted to a slow-exchange binding model to extract K_d values for NTA-Co^{2+} (c) and Co^{2+} (d) binding.

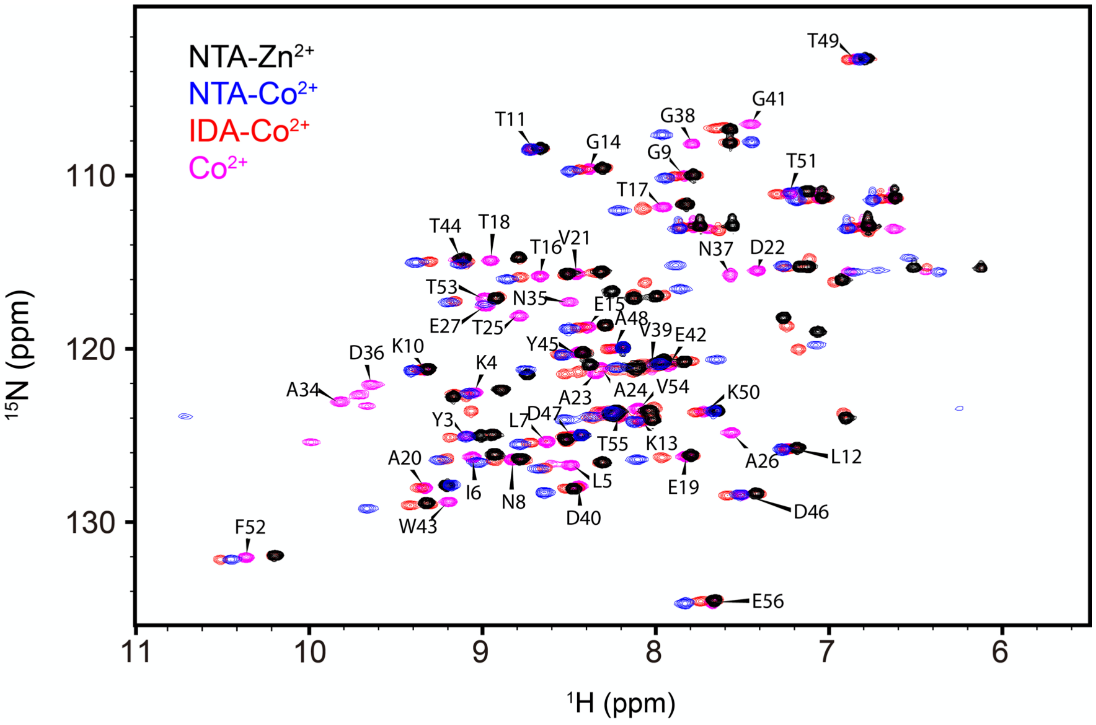

Figure S5. Superposition of ^{1}H-^{15}N HSQC spectra of diHis GB1 (0.2 mM in 20 mM HEPES, 150 mM NaCl, pH 7.0) at 283 K in the presence of saturating amounts of NTA-Zn^{2+} (black), Co^{2+}(magenta), IDA-Co^{2+} (red) or NTA-Co^{2+} (blue). The resonances between 9.5-10.0 ppm (^{1}H) in the spectrum of Co^{2+}-diHis GB1 are associated with residues near His28 and His32 and therefore exhibit large PCSs.

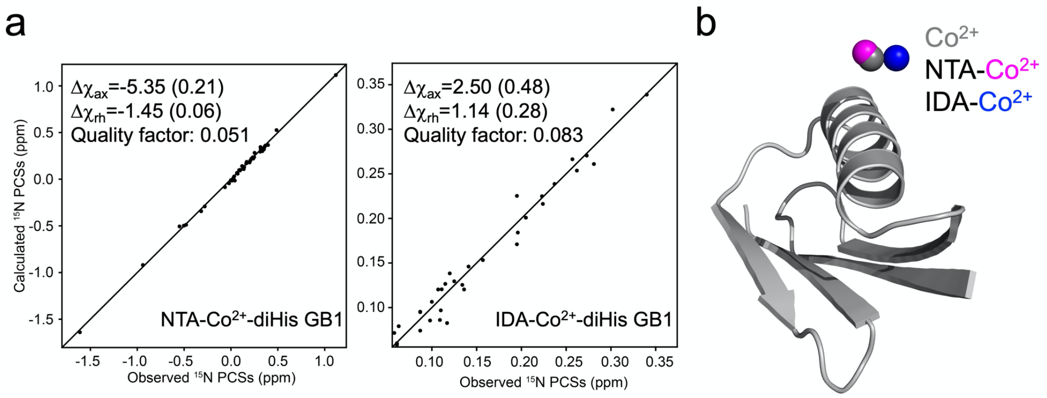

Figure S6. ^{15}N PCS analysis for NTA-Co^{2+} and IDA-Co^{2+} coordinated diHis GB1 and positions of Co^{2+} for free and capped metal ions. (a) Correlation between experimental and calculated ^{15}N PCSs. (b) Co^{2+}positions derived from ^{1}H PCSs for free Co^{2+} (grey), IDA-Co^{2+} (blue) and NTA-Co^{2+} (magenta).

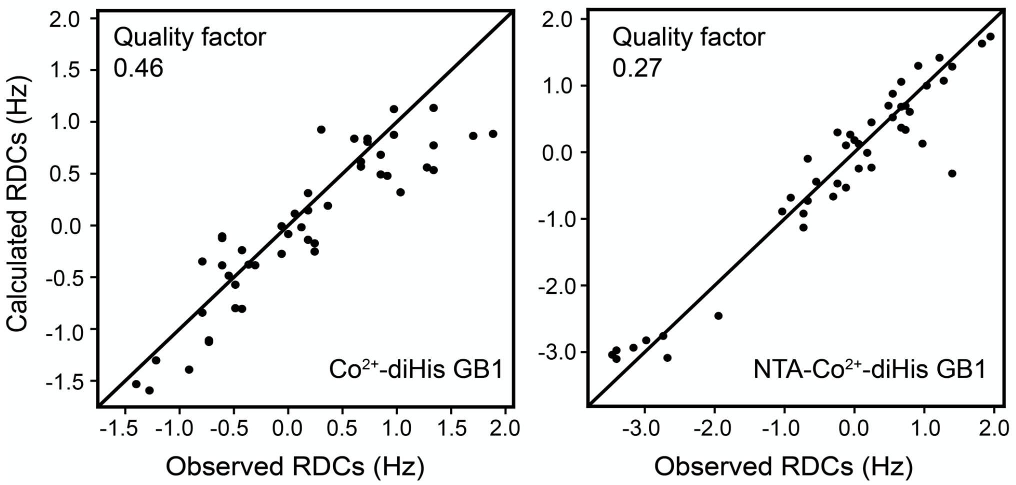

Figure S7. Correlation of measured and calculated $^{1}D_{HN}$ RDCs for Co^{2+} (left) and NTA-Co^{2+} (right) coordinated diHis GB1.

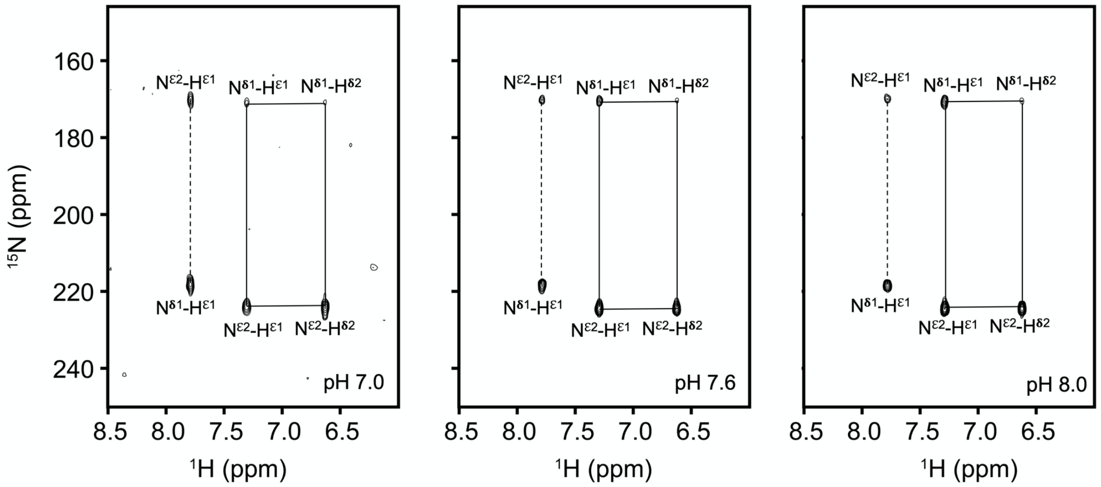

Figure S8. Long-range ^{1}H-^{15}N HMBC spectra of NTA-Zn^{2+}-diHis GB1 at pH 7.0 (left), pH 7.6 (middle) and pH 8.0 (right) in 20 mM HEPES, 150 mM NaCl buffer at 283 K.

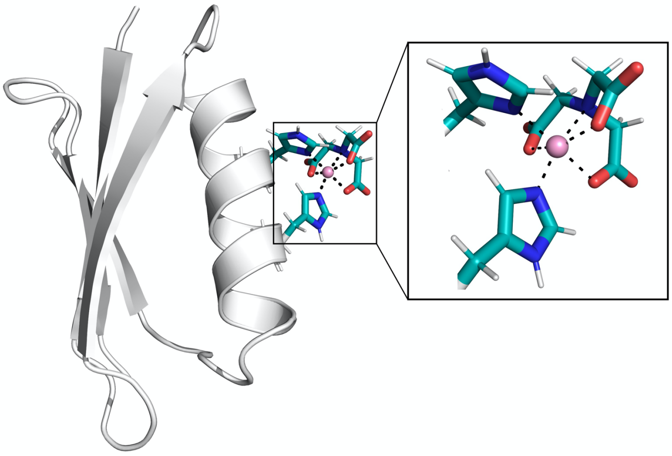

Figure S9. DFT-optimized AF2 diHis GB1 structure coordinated with NTA-Zn^{2+}. Histidine sidechains are shown in stick representation with H28 and H32 as the $N_{\varepsilon 1}$-H and $N_{\delta 1}$-H tautomers, respectively, and the cobalt ion as a pink sphere. The coordination geometry of NTA-Zn^{2+} with two imidazoles is enlarged in the far right box.

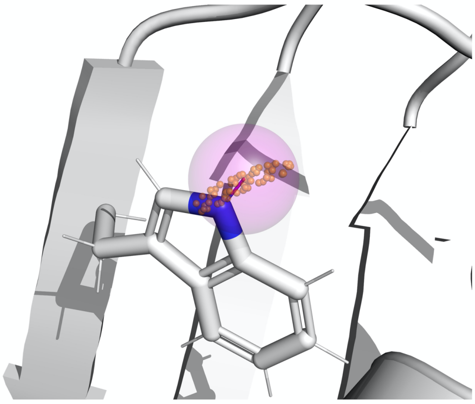

Figure S10. The plausible localization space (0.7 Å^3 volume; orange dots) of the $NH_{\varepsilon 1}$ atom of Trp43 in AF2 diHis GB1, defined by the errors of the three different Δχ-tensors for Co^{2+}, NTA-Co^{2+} and IDA-Co^{2+}-tagged GB1 and the experimental PCSs measured for $NH_{\varepsilon 1}$ atom, was calculated assuming a PCS RMSD of 0.02 ppm in Paramagpy. A magenta sphere with a radius of 1.2 Å centered at $NH_{\varepsilon 1}$ atom is shown for illustrative purposes.

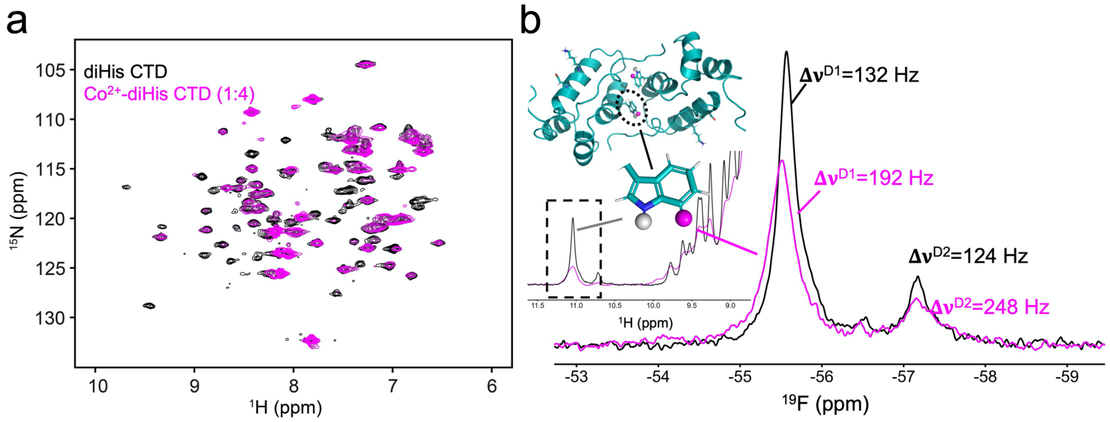

Figure S11. Addition of a four-fold excess of $CoCl_2$ to 163 μM (monomer concentration) diHis HIV CA CTD dimer. Superposition of ^{1}H-^{15}N HSQC spectra (a) and 1D ^{19}F and ^{1}H spectra (b) recorded in the absence or presence of $CoCl_2$ (at 1:4 molar ratio). The structure of the HIV CA CTD dimer (PDB id: 2KOD) is shown in ribbon representation (teal) with the Trp184 side chain in stick representation. The indole ring of Trp 184 with the NεH and 7-F atoms shown as white and magenta spheres, respectively, is enlarged below the structure. The linewidths (Δν) were determined from Lorentzian line shape fitting.

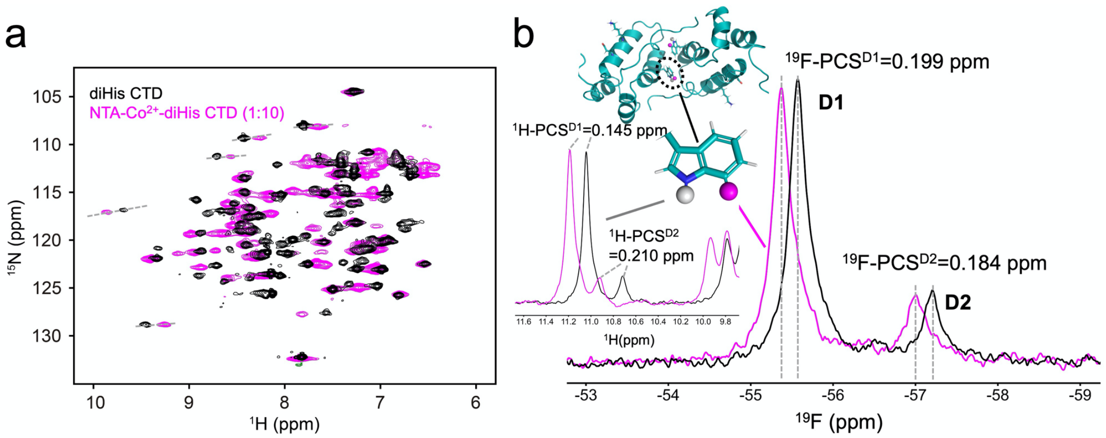

Figure S12. Addition of NTA-Co^{2+} to the diHis HIV CA CTD dimer (163 µM monomer concentration). Superposition of ^{1}H-^{15}N HSQC spectra (a) and 1D ^{19}F and ^{1}H spectra (b) recorded in the absence or presence of 10-eq NTA-Co^{2+} (mixed at 1.5:1 ratio). The structure of the CA CTD dimer (PDB id: 2KOD) is shown in ribbon representation (teal) with the Trp184 side chain in stick representation. The indole ring of Trp184 with the NεH and 7-F atoms shown as white and magenta spheres, respectively, is enlarged below the structure. ^{1}H and ^{19}F PCSs for D1 and D2 conformation are indicated in (b).

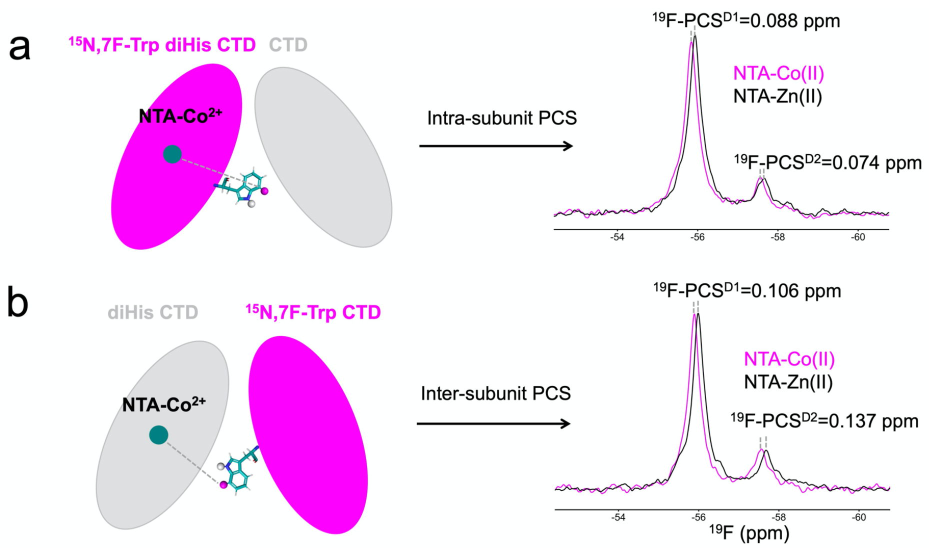

Figure S13. Schematic depiction of mixed labeled diHis CA CTD dimers for intra- and inter-subunit PCS measurements. The ^{15}N,7F-Trp184 labeled CTD chain is shown in magenta and the natural abundance (^{14}N) CTD chain is shown in grey. (a) For measuring intra-subunit PCS, the ^{15}N,7F-Trp184 diHis CTD dimer (200 μM monomer concentration) was complexed with NTA-Co^{2+} (1:10), and mixed with natural abundance CTD dimer at 1:10 ratio. (b) For measuring inter-subunit PCS, the natural abundance ^{14}N diHis CTD dimer was complexed with NTA-Co^{2+} (1:10), and mixed with ^{15}N,7F-Trp184 diHis CTD protein (100 μM monomer concentration) at 10:1 ratio.

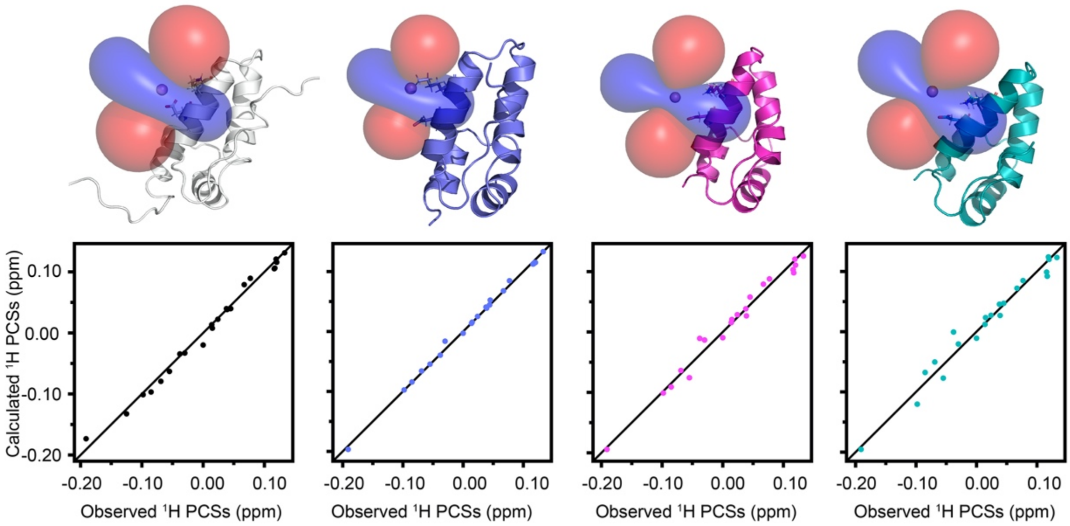

Figure S14. PCS iso-surfaces of NTA-Co^{2+} coordinated diHis CA CTD. Top panel, PCS isosurfaces of +/- 1.0 ppm around Co^{2+} for different CTD structures (PDB id: 2KOD, 4PIY, 1A43 and 1A8O) using the same color scheme as in Figure 4. Bottom, Correlation between experimental and calculated intra-subunit ^{1}H PCSs using different PDB structure.

Table S1. 5-F Trp R_2 rates for diHis GB1 and Zn^{2+}, Co^{2+}, NTA-Zn^{2+} and NTA-Co^{2+} coordinated protein (0.2 mM in 20 mM HEPES, 150 mM NaCl, pH 7.0 at 283 K).[a]

	F R_2 (s^{-1})				
diHis GB1	no metal	plus Zn^{2+}	plus Co^{2+}	plus NTA-Zn^{2+}	plus NTA-Co^{2+}
	38.0 (1.0)	78.4 (1.2)	43.3 (1.8)	37.8 (1.0)	40.8 (2.4)

[a] The fitting errors for ^{19}F R_2 are shown in the brackets.

Table S2. Fitted Δχ-tensor parameters and quality factors extracted from amide ^{1}H and ^{15}N PCSs (ppm) by Paramagpy for different dHis-GB1 structures coordinated with Co^{2+}

Structure	$\Delta\chi_{ax}$	$\Delta\chi_{rh}$	Quality factor	
	10^{-32} m^3	10^{-32} m^3	^{1}H	^{15}N*
2GB1	-3.71	-0.21	0.11	0.22 (0.08)
2QMT	-3.27	-0.01	0.06	0.16 (0.06)
1PGA	-3.22	-0.02	0.05	0.17 (0.06)
AF2 diHis GB1	-3.25	-0.07	0.05	0.17 (0.06)

*The quality factor in the brackets correspond to the fit after excluding data points colored in red in Figure 1c.

Table S3. Experimental backbone amide ^{1}H and ^{15}N PCSs (ppm) for Co^{2+}, NTA-Co^{2+} and IDA-Co^{2+} coordinated diHis GB1 (in 0.2 mM in 20 mM HEPES, 150 mM NaCl, pH 7.0) at 283K.

Residue	Co^{2+}		NTA-Co^{2+}		IDA-Co^{2+}*	
	^{1}H	^{15}N	^{1}H	^{15}N	^{1}H	^{15}N
Y3	0.131	0.144	0.154	0.141	0.247	0.143
K4	0.156	0.164	0.186	0.218	0.269	0.259
L5	0.185	0.175	0.370	0.367	0.329	0.303
I6	0.130	0.133	0.322	0.318	0.277	0.275
L7	0.112	0.116	0.270	0.312	0.199	0.221
N8	0.058	0.050	0.244	0.224	0.155	0.087
G9	0.059	0.045	0.160	0.163	0.115	0.048
K10	0.029	0.042	0.093	0.097	0.049	0.020
T11	0.038	0.047	0.060	0.064	0.029	0.050
L12	0.056	0.040	0.085	0.095	0.059	0.068
K13	0.063	0.125	0.103	0.119	0.071	0.072
G14	0.084	0.059	0.187	0.168	0.137	0.101
E15	0.101	0.110	0.215	0.238	0.149	0.127
T16	0.160	0.163	0.349	0.352	0.268	0.232
T17	0.132	0.165	0.393	0.358	0.243	0.246
T18	0.164	0.153	0.333	0.395	0.313	0.173
E19	0.035	0.060	0.311	0.247	0.167	0.180
A20	0.153	0.137	-0.020	-0.001	0.161	0.191
V21	0.148	0.127	-0.429	-0.318	0.004	0.090
D22	0.283	0.228	-0.589	-0.503	0.115	0.103
A23	0.245	0.250	-0.464	-0.479		
A24	0.363	0.415	-0.881	-0.940		
T25	0.639	0.924	-1.407	-1.612		
A26	0.640	0.920	-0.658	-0.549		
A34	0.696	0.625	1.553	1.119		
N35	0.600	0.381	0.996	0.486		
D36	0.847	0.668	0.086	-0.280		
N37	0.440	0.492	0.013	-0.021		
G38	0.222	0.123	-0.124	-0.064		
V39	0.200	0.341	0.145	0.130		
D40	-0.002	0.015	0.169	0.206	0.044	0.032
G41	-0.140	-0.073	0.392	0.327	0.078	0.031
E42	-0.052	0.025	0.268	0.363		
W43	-0.110	-0.134	0.345	0.335	0.101	0.087
T44	0.018	-0.041	0.277	0.241	0.199	0.140
Y45	0.028	0.002	0.116	0.136	0.133	0.085
D46	0.083	0.058	0.092	0.071	0.167	0.103
D47	0.058	0.065	0.003	0.041		
A48	0.043	0.012	0.002	0.011	0.080	0.037
T49	0.057	0.043	0.032	0.028	0.098	0.065
K50	0.080	0.057	0.024	0.034	0.123	0.081
T51	0.102	0.108	0.083	0.087	0.176	0.186
F52	0.167	0.149	0.255	0.233	0.314	0.253
T53	0.075	0.017	0.272	0.252	0.238	0.193
V54	0.072	-0.047	0.337	0.315		
T55	-0.007	0.256	0.299	0.448	0.137	
E56	0.020	0.105	0.173	0.198	0.083	0.090

*There are fewer assignments for IDA-Co^{2+} due to the weak binding to diHis GB1. Residues close to the paramagnetic metal ion are broadened beyond detection.

Table S4. Comparison of $\Delta\chi$-tensor parameters derived from $^{1}D_{HN}$ RDCs (measured at 600 MHz) and ^{1}H PCSs for Co^{2+} and NTA-Co^{2+} coordinated AF2 diHis-GB1 using Paramagpy.

Coordinated diHis GB1		$\Delta\chi_{ax}$ (10^{-32} m^3)	$\Delta\chi_{rh}$ (10^{-32} m^3)	α/°	β/°	γ/°
Co^{2+}	^{1}H PCSs	-3.30 (0.10)	-0.08 (0.03)	103 (1)	61 (1)	21 (44)
	$^{1}D_{HN}$ RDCs	-2.75 (0.13)	-0.78 (0.17)	107 (2)	62 (3)	49 (7)
NTA-Co^{2+}	^{1}H PCSs	-5.80 (0.34)	-1.33 (0.11)	18 (1)	103 (1)	122 (3)
	$^{1}D_{HN}$ RDCs	-5.24 (0.13)	-1.20 (0.17)	15 (1)	106 (1)	111 (4)

Table S5. Experimental intra- and inter-subunit amide ^{1}H PCSs (ppm) for the mixed labeled samples of 7F-Trp diHis CA CTD dimers (see Fig. S8; 0.2 mM, 20mM HEPES, 150 mM NaCl, 1mM TCEP, pH 7.0) at 283K.

Measured Intra-subunit PCSs		Measured Inter-subunit PCSs	
Residue index	ppm	Residue index	ppm
146	-0.023	148	0.012
148	0.044	151	0.030
156	0.120	153	0.050
158	0.010	155	0.032
159	-0.030	156	0.019
161	-0.191	158	0.054
176	-0.055	159	0.029
177	-0.038	161	0.017
178	0.014	162	0.022
180	0.039	163	0.006
182	0.077	165	0.015
183	0.116	167	0.010
184	0.117	168	0.018
194	0.133	169	0.005
195	0.067	172	0.030
205	0.015	174	0.025
206	0.024	176	0.038
209	0.045	177	0.043
210	0.038	178	0.047
211	0.119	179	0.043
216	-0.098	180	0.034
217	-0.069	181	0.051
218	-0.085	182	0.030
223	-0.125	183	0.025
		184	0.027
		186	0.037
		189	0.053
		193	0.043
		194	0.035
		195	0.025
		197	0.020
		198	0.021
		199	0.024
		201	0.013
		202	0.015

Table S6. Fitted $\Delta\chi$-tensor parameters and quality factors generated by Paramagpy from intra-subunit ^{1}H PCSs (ppm) for different dimer structures of NTA-Co^{2+}-dHis CA CTD. [a]

Structure	$\Delta\chi_{ax}$	$\Delta\chi_{rh}$	Quality factor
	10^{-32} m^3	10^{-32} m^3	^{1}H
2KOD	-4.29 (0.94)	-0.81 (0.22)	0.10
4PIY	-4.13 (0.37)	-0.74 (0.10)	0.06
1A8O	-3.11 (0.49)	-1.40 (0.26)	0.17
1A43	-2.12 (0.42)	-1.19 (0.20)	0.14

[a] The fitting errors for the tensor parameters are shown in the brackets.

Table S7. Experimental and back-calculated ^{1}H and ^{19}F PCSs (ppm) for Trp184 for different CA CTD dimers (D1) with the $\Delta\chi$-tensor parameters of Table S6.

Atom position in Trp184	Measured PCSs (ppm)	Calculated PCSs (ppm) PDB id				
	D1	4IPY	2KOD	1A8O	1A43	AF2 CTD
intra_HZ2 (7F)	0.088	0.092	0.066	0.034	0.039	0.081
inter_HZ2 (7F)	0.106	0.099	0.076	0.159	0.034	0.093
inter_HE1	0.040	0.050	0.045	0.129	0.028	0.050
intra+inter_HE1	0.145	0.131	0.108	0.18	0.071	0.123
intra+inter_HZ2 (7F)	0.199	0.191	0.153	0.202	0.073	0.174

References

1. Byeon, I. J.; Meng, X.; Jung, J.; Zhao, G.; Yang, R.; Ahn, J.; Shi, J.; Concel, J.; Aiken, C.; Zhang, P.; Gronenborn, A. M., Structural Convergence between Cryo-EM and NMR Reveals Intersubunit Interactions Critical for HIV-1 Capsid Function. *Cell* **2009,** *139* (4), 780-90.

2. Cordier, F.; Dingley, A. J.; Grzesiek, S., A doublet-separated sensitivity-enhanced HSQC for the determination of scalar and dipolar one-bond J-couplings. *J. Biomol. NMR.* **1999**, 13 (2), 175-180.

3. Lee, W.; Tonelli, M.; Markley, J. L., NMRFAM-SPARKY: enhanced software for biomolecular NMR spectroscopy. *Bioinformatics.* **2015**, 31 (8), 1325-7.

4. Orton, H. W.; Huber, T.; Otting, G., Paramagpy: Software for Fitting Magnetic Susceptibility Tensors Using Paramagnetic Effects Measured in NMR Spectra. *Magn. Reson.* **2020,** *1* (1), 1-12.

5. Vold, R. L., On the Measurement of Transverse Relaxation Rates in Complex Spin Systems. *J. Chem. Phys.* **1972,** *56* (7), 3210-3216.

6. Carr, H. Y.; Purcell, E. M., Effects of Diffusion on Free Precession in Nuclear Magnetic Resonance Experiments. *Phys. Rev.* **1954,** *94* (3), 630-638.

7. Meiboom, S.; Gill, D., Modified Spin-Echo Method for Measuring Nuclear Relaxation Times. *Rev. Sci. Instrum.* **1958,** *29* (8), 688-691.

8. Luz, Z.; Meiboom, S., Nuclear Magnetic Resonance Study of the Protolysis of Trimethylammonium Ion in Aqueous Solution—Order of the Reaction with Respect to Solvent. *J. Chem. Phys.* **1963,** *39* (2), 366-370.

9. Lee, D.; Hilty, C.; Wider, G.; Wüthrich, K., Effective Rotational Correlation Times of Proteins from NMR Relaxation Interference. *J. Magn. Reson.* **2006,** *178* (1), 72-76.

10. Orton, H. W.; Abdelkader, E. H.; Topping, L.; Butler, S. J.; Otting, G., Localising nuclear spins by pseudocontact shifts from a single tagging site. *Magn. Reson. Discuss.* **2022**, 2022, 1-21.

11. Oliver, J. D.; Barnett, B. L.; Strickland, L. C., Structures of Sodium Zinc Nitrilotriacetate Monohydrate (173 K) and Disodium Hydrogen Nitrilotriacetate (293 K). *Acta Cryst.* **1984,** *40* (4), 377-381.

12. Hanwell, M. D.; Curtis, D. E.; Lonie, D. C.; Vandermeersch, T.; Zurek, E.; Hutchison, G. R., Avogadro: An Advanced Semantic Chemical Editor, Visualization, and Analysis Platform. *J. Cheminf.* **2012,** *4* (1), 1-17.

13. Neese, F.; Wennmohs, F.; Becker, U.; Riplinger, C., The ORCA Quantum Chemistry Program Package. *J. Chem. Phys.* **2020,** *152* (22), 224108-224125.

14. Orio, M.; Pantazis, D. A.; Neese, F., Density Functional Theory. *Photosynth. Res.* **2009,** *102* (2), 443-453.

15. Becke, A. D., Density-functional Exchange-energy Approximation with Correct Asymptotic Behavior. *Phys. Rev. A* **1988,** *38* (6), 3098-3100.

16. Whitten, J. L., Coulombic Potential Energy Integrals and Approximations. *J. Chem. Phys.* **1973,** *58* (10), 4496-4501.

17. Grimme, S.; Antony, J.; Ehrlich, S.; Krieg, H., A Consistent and Accurate *ab initio* Parametrization of Density Functional Dispersion Correction (DFT-D) for the 94 Elements H-Pu. *J. Chem. Phys.* **2010,** *132* (15), 154104-154122.

18. Weigend, F.; Ahlrichs, R., Balanced Basis Sets of Split Valence, Triple Zeta Valence and Quadruple Zeta Valence Quality for H to Rn: Design and Assessment of Accuracy. *Phys. Chem. Chem. Phys.* **2005,** *7* (18), 3297-3305.

19. Marenich, A. V.; Cramer, C. J.; Truhlar, D. G., Universal Solvation Model Based on Solute Electron Density and on a Continuum Model of the Solvent Defined by the Bulk Dielectric Constant and Atomic Surface Tensions. *J. Phys. Chem. B* **2009,** *113* (18), 6378-6396.

20. Pettersen, E. F.; Goddard, T. D.; Huang, C. C.; Couch, G. S.; Greenblatt, D. M.; Meng, E. C.; Ferrin, T. E., UCSF Chimera—A Visualization System for Exploratory Research and Analysis. *J. Comput. Chem.* **2004,** *25* (13), 1605-1612.

21. Visser, H. G.; Purcell, W.; Basson, S. S., A New Reaction Route for the Synthesis of Different Cobalt(III) Nitrilotriacetato Complexes. The Crystal Structure of $Cs_2[Co(nta)(CO_3)]\cdot H_2O$. *Polyhedron* **2001,** *20* (3), 185-190.

22. Bogetti, X.; Ghosh, S.; Gamble Jarvi, A.; Wang, J.; Saxena, S., Molecular Dynamics Simulations Based on Newly Developed Force Field Parameters for Cu^{2+} Spin Labels Provide Insights into Double-Histidine-Based Double Electron–Electron Resonance. *J. Phys. Chem. B* **2020,** *124* (14), 2788-2797.

23. Case, D. A.; Cheatham III, T. E.; Darden, T.; Gohlke, H.; Luo, R.; Merz Jr., K. M.; Onufriev, A.; Simmerling, C.; Wang, B.; Woods, R. J., The Amber Biomolecular Simulation Programs. *J. Comput. Chem.* **2005,** *26* (16), 1668-1688.

24. Yang, D. T.; Gronenborn, A. M.; Chong, L. T., Development and Validation of Fluorinated, Aromatic Amino Acid Parameters for Use with the AMBER ff15ipq Protein Force Field. *J. Phys. Chem. A* **2022,** *126* (14), 2286-2297.

25. Debiec, K. T.; Cerutti, D. S.; Baker, L. R.; Gronenborn, A. M.; Case, D. A.; Chong, L. T., Further along the Road Less Traveled: AMBER ff15ipq, an Original Protein Force Field Built on a Self-Consistent Physical Model. *J. Chem. Theory Comput.* **2016,** *12* (8), 3926-3947.

26. Wang, J.; Wolf, R. M.; Caldwell, J. W.; Kollman, P. A.; Case, D. A., Development and Testing of a General Amber Force Field. *J. Comput. Chem.* **2004,** *25* (9), 1157-1174.

27. Wang, J.; Wang, W.; Kollman, P. A.; Case, D. A., Automatic Atom Type and Bond Type Perception in Molecular Mechanical Calculations. *J. Mol. Graphics Modell.* **2006,** *25* (2), 247-260.

28. Li, P.; Merz, K. M., Taking into Account the Ion-Induced Dipole Interaction in the Nonbonded Model of Ions. *J. Chem. Theory Comput.* **2014,** *10* (1), 289-297.

29. Takemura, K.; Kitao, A., Water Model Tuning for Improved Reproduction of Rotational Diffusion and NMR Spectral Density. *J. Phys. Chem. B* **2012,** *116* (22), 6279-6287.

30. Joung, I. S.; Cheatham, T. E., Determination of Alkali and Halide Monovalent Ion Parameters for Use in Explicitly Solvated Biomolecular Simulations. *J. Phys. Chem. B* **2008,** *112* (30), 9020-9041.

31. Essmann, U.; Perera, L.; Berkowitz, M. L.; Darden, T.; Lee, H.; Pedersen, L. G., A Smooth Particle Mesh Ewald Method. *J. Chem. Phys.* **1995,** *103* (19), 8577-8593.

32. Ryckaert, J.-P.; Ciccotti, G.; Berendsen, H. J. C., Numerical Integration of the Cartesian Equations of Motion of a System with Constraints: Molecular Dynamics of N-alkanes. *J. Comput. Phys.* **1977,** *23* (3), 327-341.
